# Tender cervical lymphadenitis as a herald of multi-system inflammatory syndrome in COVID-19 infection of children and adolescents: a report of two cases

**DOI:** 10.1186/s12879-022-07943-w

**Published:** 2022-12-16

**Authors:** Leonard Wanninayake, Gayathree de Abrew, Dinusha Logeshwaran, Chathurika Weerasinghe, Piyumi Gowinna, Sachith Mettananda, Ranjan Premaratna

**Affiliations:** 1 University Medical Unit, Colombo North Teaching Hospital, Ragama, Sri Lanka; 2 Department of Pharmacology, Faculty of Medicine, University of Colombo, Colombo, Sri Lanka; 3 University Paediatric Unit, Colombo North Teaching Hospital, Ragama, Sri Lanka; 4 Department of Paediatrics, Faculty of Medicine, University of Kelaniya, Ragama, Sri Lanka; 5 Department of Medicine, Faculty of Medicine, University of Kelaniya, PO Box 6, Thalagolla Road, Ragama, Sri Lanka

**Keywords:** Multisystem inflammatory syndrome, Cervical lymphadenitis, COVID 19, Case report

## Abstract

**Background:**

Post-COVID-19 multisystem inflammatory syndrome (MIS) has been increasingly recognized but fever with isolated tender cervical lymphadenitis as the initial presentation has been rarely reported. We present 2 female patients one a child and the other an adolescent.

**Case presentation:**

Case 1 was a 13-year-old girl who presented with tender cervical lymphadenopathy and fever 3-weeks post-COVID-19, and then developed features of MIS 5 days later. Case 2, also female, was 18 years old. She had no history of COVID-19 infection or immunization but had a serologic diagnosis of COVID-19. She similarly presented with fever and tender cervical lymphadenopathy, and then progressed rapidly to develop features of MIS. Both patients responded well to treatment with immunosuppressants and intravenous immunoglobulin.

**Conclusion:**

Tender cervical lymphadenopathy could be the herald of multi-system inflammatory syndrome following COVID-19 infection among children and adolescents, which the clinicians must have a good suspicion about.

## Introduction

Multi system inflammatory syndrome (MIS) following COVID 19 infection is still under study. It is mainly described in children (MIS-C) [1], however, recently a similar clinical entity has been reported in adults (MIS-A) [1]. MIS-C is described like Kawasaki disease or toxic shock syndrome. Its presentations vary and include persistent fever and combinations of symptoms related to gastrointestinal, cardiovascular, neurological, or haematological involvement. Severe most illness may lead to haemodynamic instability and development of shock. Elevated inflammatory markers and high antibody titres against COVID 19 infection helps in the diagnosis [2, 3]. MIS also known to result in several dermatological manifestations and lymphadenopathy [1, 3]. Recently reported case series of MIS-A highlight fever being the most common symptom and is documented in 96% of the patients [3]. Other reported clinical features are related to haematological involvement in 92%, hypotension in 60%, cardiac dysfunction in 54%, diarrhoea in 52% and the shortness of breath in 52% [1, 3–5].

In pre COVID 19 era, acute fever with tender cervical lymphadenopathy has been a common clinical presentation where viral or bacterial aetiologies are considered and mostly managed as outpatients [6, 7]. Although occurrence of lymphadenopathy together with symptoms such as conjunctival injection, skin rashes, vomiting, diarrhoea together with features of cardiovascular involvement and hypotension has been described in MIS [3] However, initial presentation with tender cervical lymphadenitis and fever has been rare [8]. We present 2 female adolescents who had fever with isolated tender cervical lymphadenitis as the initial presentation of MIS.

## Case 1

13-year-old healthy girl who had COVID-19 3 weeks before, had presented to the general practitioner with fever and painful right sided neck swelling for 3 days and received oral clarythromycin treatment based on high WBC (11.5 × 10^3^) and c-reactive protein [CRP] (76 mg/dL). However, as there was no response, she presented to specialist care on the 5th day of illness with worsening symptoms. Examination revealed tender right cervical lymphadenitis with no tonsillar or pharyngeal inflammation. There were few 1–3 mm blisters on the trunk. After 2 days, she developed conjunctivitis, vomiting, diarrhoea and generalized erythematous macular rash and erythematous mucous membranes. Her pulse rate was 120/min and BP was 80/60 mmHg.

There was neutrophil leucocytosis with elevated CRP, ESR, serum ferritin, LDH, AST and ALT (Table 1), but serum procalcitonin and blood cultures were negative. Chest X-ray was normal, ECG showed sinus tachycardia and 2D transthoracic echo cardiogram showed a structurally normal heart with prominent and mildly dilated coronary arteries with no ectatic segments or aneurysms. The troponin I was normal. Anti-COVID 19 IgM and IgG were positive in very high titres. Her investigations on admission are summarized in Table 1. Based on above features, a clinical diagnosis of severe MIS-C was made.

**Table 1:** Investigations of case 1 and case 2

Investigation	Results of case 1	Results of case 2
Haemoglobin	13.4 g/dL [12–15]	12.8 g/dL [12–15]
White cell count (WBC)	11.5 × 10^3^ [4–11 × 10^3^]	6.06 × 10^3^ [4–11 × 10^3^]
Neutrophils	88%	91%
Lymphocytes	10%	7%
Platelets	105 × 10^3^ [150–450 × 10^3^].	79 × 10^3^ [150–450 × 10^3^]
C-Reactive Protein (CRP)	108 mg/dL [0–5]	CRP 311 mg/L [0–5],
Erythrocyte sedimentation rate [ESR]	38 mm/1st hour [< 20]	58 mm/1st hour [< 20]
Aspartate transaminase [AST]	200 U/L [< 35]	79 U/L [< 35]
Alanine transaminase [ALT	133 U/L [< 35]	76 U/L [< 35]
Lactate dehydrogenase [LDH]	573 U/L [< 250]	260 U/L [< 250]
Troponin I	0.051 [< 0.012]	0.03 [< 0.012]
Ferritin	498.2 ng/mL [9.3–159]	287 ng/mL [9.3–153]
D dimer	7.76 mg/L [< 0.5]	5260 mg/mL [< 0.5]
Procalcitonin	0.2 ng/mL [< 0.5]	2.5 ng/mL [< 0.5]
Blood culture	No growth	No growth
Sputum culture	No growth	No growth
COVID 19 IgM antibody titre	2.8 [< 0.9 to > 1.1],	3.7 [< 0.9 to > 1.1]
COVID-19 IgG antibody titre	28.9 [< 0.9 to > 1.1]	23 [< 0.9 to > 1.1]
ECG	Sinus tachycardia	Sinus tachycardia
2D Echocardiogram	Structurally normal heart with prominent and mildly dilated coronary arteries with no ectatic segments or aneurysms	Structurally normal heart with prominent and mildly dilated coronary arteries but no ectatic segments or aneurysms
HRCT	Not performed	Extensive consolidation with ground-glass opacities in dependant areas of bilateral lungs
CMV IgM antibodies	Negative	Not done
IMN IgM antibodies	Negative	
ANA	1: 8	Not done

*HRCT* high resolution computed tomography, *CMV* cytomegalovirus, *IMN* infectious mononucleosis, *ANA* anti-nuclear antibodies

She was treated with intravenous fluid boluses, inotropic support [dobutamine], and the specific management was based on the updated clinical consensus on the management of MIS-C [6].

## Case 2

A 18-year-old otherwise healthy schoolgirl had a similar presentation to primary care with painful right sided neck lump for 4 days and high-grade intermittent fever with chills for 2 days. She had a mild cough with scanty non-purulent sputum. She did not have a history or contact with COVID 19 infection and did not receive vaccination against COVID 19. She had received treatment with oral antibiotics as for bacterial lymphadenitis. One day later she presented for specialist care with worsening symptoms and vomiting. She also had a pulse rate of 110/min and blood pressure BP 85/60 mmHg.

She had thrombocytopaenia with neutrophil predominance, elevated CRP, ferritin, LDH and D dimer. However, procalcitonin and blood cultures were negative. ECG showed sinus tachycardia and Trop I was normal. 2D Echo showed structurally normal heart with prominent and mildly dilated coronary arteries but no ectatic segments or aneurysms like first patient. She developed low arterial O_2_ saturation (90%) on air on third day warranting supplementary O_2_ via nasal prongs. Chest X-ray was normal and there were no inflammatory shadows or pleural effusion. CTPA which was negative for pulmonary embolism. HRCT showed evidence of extensive consolidation with ground-glass opacities in dependant areas of both lungs suggesting MIS associated with COVID 19 [7]. Her investigations are summarized in Table 1.

A clinical diagnosis of MIS was made based on the multisystem involvement with strongly positive COVID 19 serology. Patient was given ICU care for 3 days and specific management was commenced like for the first patient and the heart rate was controlled with Ivabradine 2.5 mg twice daily. Inotropic support with given with noradrenaline 0.1 µg/kg/h during the first 2 days of ICU care. She had a gradual improvement over 72 h. On the 7th day of illness she developed a transient erythematous, non-scaly, blanching rash in upper limbs and in the torso and this was considered an Iv-Ig induced drug reaction [8]. After 11 days of institutional care, patient had a full recovery and was discharged on tapering doses of oral steroids. After 8 weeks of treatment, she had a remarkable recovery and was in good health.

## Discussion

Both above patients, who were previously well, presented with tender cervical lymphadenitis and rapidly progressed to involve other systems. The recent history of COVID-19 in the first patient together with few skin blisters, gastrointestinal and cardiovascular findings prompted the possibility of MIS. Although, the second patient had no history of COVID-19 or contact history, its likely her initial symptoms could have been overlooked at primary contact level and/or she had contacted the illness from an asymptomatic patient with COVID-19. However, it’s the experience of the first patient that helped in the diagnosis of the second patient, otherwise the illness of this would have been missed leading to a catastrophic ending.

In a recent publication, a 28-year-old man who has had fever and right sided submandibular lymphadenopathy was diagnosed having MIS-A by excluding all other possible aetiologies [5]. However, patient had no other systemic manifestations and has improved without immunosuppressive treatment [5]. Our second patient is 18 years, her illness would probably fall into MIS-A and she had an acute illness together with systemic involvement suggested by the tachycardia, hypotension, transaminitis and the lung involvement. She warranted immune-suppressive treatment; however, the treatment was based on MIS-C management guidelines [6] as there was no clear consensus in the management of MIS-A.

Fever with lymphadenitis is a common clinical presentation among children and adolescents. Common causes of such presentation include infectious mononucleosis, CMV infection, HIV, streptococcal pharyngitis, or tonsillitis, SLE and tuberculosis. However, the first three illnesses are viral aetiologies and results in normal inflammatory markers such as normal CRP together with reactive lymphocytosis in the blood picture. In bacterial infections, high inflammatory markers with raised neutrophil counts are expected. SLE (systemic lupus erythematosus) is known to cause a low WBC, high ESR and a low CRP level. Tuberculosis would result in a non-tender lymphadenopathy and other constitutional symptoms such as loss of appetite and weight loss. Although MIS associated with COVID-19 infection is mostly an illness of exclusion of above common aetiologies, presence of skin blisters or other dermatological manifestations, and systemic manifestations such as unexplained tachycardia or low blood pressure should prompt the suspicion of MIS [1, 3, 5, 8] and investigated appropriately, as delay in treatment is likely to result in a deleterious outcome.

## Conclusion

Tender cervical lymphadenopathy could be the herald of multi-system inflammatory syndrome following COVID-19 infection among children and adolescents, which the clinicians must have a good suspicion about. As this is an emerging clinical entity during the post COVID era, that may mimic common clinical scenarios we recommend careful assessment, follow up and documentation of similar illness to facilitate detection of MIS at an early stage.

## Data Availability

Data sharing is not applicable to this article as no datasets were generated or analysed during the current study. However, all clinical materials are available with the respective patients and their bed head tickets are available in the hospital record room maintaining confidentiality.

## Abbreviations

MIS C: Multisystem inflammatory syndrome in children
MIS A: Multisystem inflammatory syndrome in adults
WBC: White blood cells
CRP: C reactive protein
BP: Blood pressure
N: Neutrophils
ESR: Erythrocyte sedimentation rate
AST: Aspartate transaminase
ALT: Alanine transaminase
LDH: Lactate dehydrogenase
ALP: Alkaline phosphatase
PCR: Polymerase chain reaction
ECG: Electrocardiogram
HRCT: High resolution computed tomogram
O_2_: Oxygen
CTPA: Computed tomogram pulmonary angiogram
AFB: Acid fast bacilli
TB: Tuberculosis
ICU: Intensive care unit
IV: Intravenous
IV Ig: Intravenous immunoglobulin
